# Supplementary material for: Library size-stabilized metacells construction enhances co-expression network analysis in single-cell data
Source: PLoS Comput Biol. 2025 Nov 13;21(11):e1013697. doi: 10.1371/journal.pcbi.1013697 (PMC12626273; doi:10.1371/journal.pcbi.1013697)
Supplement: S1 File — Contains the theoretical proof of our main results, where we establish theoretically that compositional gene expression data suffers from inflated Type I errors in correlation analyses. (PDF) [file pcbi.1013697.s001.pdf]

Supplementary Note: Proof for the Main Results

In this section, we establish theoretically that compositional gene expression data suffers from inflated Type I errors in correlation analyses. We demonstrate that an increase in the variance of latent scaling factors not only amplifies the variance of normalized gene expression correlations but also induces an upward bias. When hypothesis testing employs unadjusted critical values, this amplification systematically increases the likelihood of falsely rejecting the null hypothesis (Type I error).

Before proceeding with the main Theorem, we need the following Assumptions, which formalize the data-generating process:

Assumption **1.** *The latent total gene expression count for the k-th cell, denoted S'_k, follows a truncated normal distribution with mean μ_S, variance σ_S^2, and support $S'_k > 0$. The variables $\{S'_k\}_{k=1}^n$ are mutually independent.*

Assumption **2.** *The observed total expression count S_k in the k-th cell is a scaled version of the latent S'_k, with variance inflated by a factor $h > 1$:*

$$\mathrm{Var}(S_k) = h\sigma_S^2.$$

Furthermore, $h < h_{max} = O(1)$, for some bound h_{max}.

Assumption **3.** *Conditional on S_k, the gene-specific expression count $X_{i,k}$ for gene i in cell k follows a Poisson distribution with rate $\alpha_i S_k$, where $0 < \alpha_i < 1$ and $\sum_{i=1}^p \alpha_i = 1$. This ensures $\mathbb{E}\left[\sum_{i=1}^p X_{i,k} \mid S_k\right] = S_k$.*

Recall that our question of interest is:

"Are the expressions of two genes correlated?"

This can be formulated into a classical hypothesis testing question of

$$\mathcal{H}_0 : \rho = 0 \quad \text{vs} \quad \mathcal{H}_1 : \rho \neq 0.$$

Define the log-transformed variables as

$$Y_k = \log\left(\frac{X_{i,k}}{S_k} + 1\right), \quad Z_k = \log\left(\frac{X_{j,k}}{S_k} + 1\right).$$

Denote their Pearson correlation estimator as $\hat{\rho}$. We have the following results.

Theorem 1. *Suppose that Assumptions 1-3 hold. Under H_0, $\rho = 0$, if the library size S_k exhibits significant variability (i.e., $h > 1$), the Pearson correlation estimator $\hat{\rho}$ between log-transformed variables*

$$Y_k = \log\left(\frac{X_{i,k}}{S_k} + 1\right) \quad \textit{and} \quad Z_k = \log\left(\frac{X_{j,k}}{S_k} + 1\right)$$

has non-zero expectation, i.e., $\mathbb{E}[\hat{\rho}] \neq 0$.

Proof. First, note that the normalized counts are conditionally independent under the null,

$$\mathrm{Cov}\left(\frac{X_{i,k}}{S_k}, \frac{X_{j,k}}{S_k}\Big| S_k\right) = 0. \tag{1}$$

For $W_{i,k} \triangleq X_{i,k}/S_k$, by Taylor's expansion of $Y_k = \log(W_{i,k} + 1)$ at α_i, we have

$$Y_k = \log(\alpha_i + 1) + \frac{W_{i,k} - \alpha_i}{\alpha_i + 1} - \frac{(W_{i,k} - \alpha_i)^2}{2(\alpha_i + 1)^2} + R_3 \tag{2}$$

where $|R_3| \leq \frac{|W_{i,k} - \alpha_i|^3}{3(\alpha_i + 1)^3}$ almost surely. Taking conditional expectation of (2), we have

$$\mathbb{E}[Y_k|S_k] = \log(\alpha_i + 1) - \frac{\alpha_i}{2(\alpha_i + 1)^2 S_k} + \mathbb{E}[R_3|S_k], \tag{3}$$

where

$$\mathbb{E}[R_3|S_k] = O\left(\frac{1}{S_k^2}\right). \tag{4}$$

By the law of total covariance, we can derive that

$$\begin{aligned}\mathrm{Cov}(Y_k, Z_k) &= \mathrm{Cov}(\mathbb{E}[Y_k|S_k], \mathbb{E}[Z_k|S_k]) + \mathbb{E}[\mathrm{Cov}(Y_k, Z_k|S_k)] \\ &= \mathrm{Cov}\left(\log(\alpha_i+1) - \frac{\alpha_i}{2(\alpha_i+1)^2 S_k}, \log(\alpha_j+1) - \frac{\alpha_j}{2(\alpha_j+1)^2 S_k}\right) \end{aligned}\tag{5}$$

Applying Taylor's expansion on (5), we have

$$\mathrm{Cov}(Y_k, Z_k) = \frac{\alpha_i\alpha_j}{4(\alpha_i+1)^2(\alpha_j+1)^2}\mathrm{Var}\left(\frac{1}{S_k}\right) + O\left(\mathbb{E}\left[\frac{1}{S_k^3}\right]\right). \tag{6}$$

Similarly, the variances follow that

$$\mathrm{Var}(Y_k) = \frac{\alpha_i}{(\alpha_i+1)^2}\mathbb{E}\left[\frac{1}{S_k}\right] + O\left(\mathbb{E}\left[\frac{1}{S_k^2}\right]\right), \tag{7}$$

$$\mathrm{Var}(Z_k) = \frac{\alpha_j}{(\alpha_j+1)^2}\mathbb{E}\left[\frac{1}{S_k}\right] + O\left(\mathbb{E}\left[\frac{1}{S_k^2}\right]\right). \tag{8}$$

Hence, we have shown that

$$\mathbb{E}[\hat{\rho}] = \frac{\mathrm{Cov}(Y_k, Z_k)}{\sqrt{\mathrm{Var}(Y_k)\mathrm{Var}(Z_k)}} = \frac{\alpha_i\alpha_j}{4(\alpha_i+1)^2(\alpha_j+1)^2} \cdot \frac{\mathrm{Var}(1/S_k)}{\mathbb{E}[1/S_k]} + o(1) > 0, \tag{9}$$

which finishes the proof. □

Theorem 2. *Under the conditions of Theorem 1, the variance of the Pearson correlation estimator satisfies:*

$$\frac{\partial}{\partial h}\mathrm{Var}(\hat{\rho}) > 0 \quad \text{for } h > 1.$$

Proof. Let $S_k \sim \mathcal{N}_T(\mu_S, h\sigma_S^2)$ with truncation at $S_k > 0$. Define $\gamma \triangleq \mu_S/\sqrt{h\sigma_S^2}$. The inverse moment follows

$$\mathbb{E}[1/S_k] = \frac{1}{\sqrt{h\sigma_S^2}} \cdot \frac{\phi(-\gamma)}{1-\Phi(-\gamma)} \cdot \left(1 + \frac{\gamma}{1-\Phi(-\gamma)/\phi(-\gamma)}\right). \tag{10}$$

Through differentiation under the integral sign, we can derive that

$$\frac{\partial}{\partial h}\mathbb{E}[1/S_k] = \frac{\mu_S^2}{2h^{3/2}\sigma_S^3} \cdot \frac{\phi(-\gamma)}{[1-\Phi(-\gamma)]^2} > 0. \tag{11}$$

For $W_{i,k} \triangleq X_{i,k}/S_k$, using the law of total variance, we have

$$\mathrm{Var}(W_{i,k}) = \mathbb{E}[\mathrm{Var}(W_{i,k}|S_k)] + \mathrm{Var}(\mathbb{E}[W_{i,k}|S_k]) = \alpha_i\mathbb{E}[1/S_k] + 0. \tag{12}$$

For $Y_k = g(W_{i,k}) = \log(W_{i,k}+1)$, by Taylor's expansion to the second order, we have

$$Y_k = g(\alpha_i) + g'(\alpha_i)(W_{i,k}-\alpha_i) + \frac{1}{2}g''(\tilde{W}_{i,k})(W_{i,k}-\alpha_i)^2 \tag{13}$$

where $\tilde{W}_{i,k}$ lies between $W_{i,k}$ and α_i. Taking variance, (13) implies

$$\mathrm{Var}(Y_k) = \frac{\alpha_i}{(\alpha_i+1)^2}\mathbb{E}[1/S_k] + \frac{1}{4}\mathbb{E}\left[\frac{(W_{i,k}-\alpha_i)^4}{(\tilde{W}_{i,k}+1)^4}\right]. \tag{14}$$

From (11) and (12), the leading term in (14) satisfies

$$\frac{\partial}{\partial h}\left(\frac{\alpha_i}{(\alpha_i+1)^2}\mathbb{E}[1/S_k]\right) > 0. \tag{15}$$

The remainder term in (14) is bounded by

$$\left|\frac{1}{4}\mathbb{E}\left[\frac{(W_{i,k}-\alpha_i)^4}{(\tilde{W}_{i,k}+1)^4}\right]\right| \leq \frac{3\alpha_i^2+\alpha_i}{4(\alpha_i+1)^4}\mathbb{E}[1/S_k^2] = O(h^{-1}).$$

Next, recall the asymptotic distribution of Pearson correlation

$$\mathrm{Var}(\hat{\rho}) = \frac{(1-\rho^2)^2}{n} + \frac{\rho^2}{2n}\left(\frac{\mathrm{Var}(Y_k)}{\sigma_Y^2} + \frac{\mathrm{Var}(Z_k)}{\sigma_Z^2}\right) + o(n^{-1}). \tag{16}$$

Substituting $\rho = 0$ and $\partial \mathrm{Var}(Y_k)/\partial h > 0$ from (15), we finally have

$$\frac{\partial}{\partial h}\mathrm{Var}(\hat{\rho}) = \frac{1}{2n}\left(\frac{\partial \mathrm{Var}(Y_k)}{\sigma_Y^2} + \frac{\partial \mathrm{Var}(Z_k)}{\sigma_Z^2}\right) + o(n^{-1}) > 0.$$

This implies that the asymptotic variance of the estimated Pearson correlation increases as the scaling factor $h > 1$ increases. □

Corollary **1.** *Under the same conditions in Theorems 1-2, for any nominal level $\alpha \in (0,1)$, the actual Type I error rate satisfies:*

$$P(|\hat{\rho}| > c_\alpha \mid H_0) > \alpha,$$

where $c_\alpha = \Phi^{-1}(1-\alpha/2)\sigma$ is the critical value calculated under $\mathcal{H}_0$.

Proof. For $W = \frac{X_{i,k}}{S_k} \sim \mathrm{Poisson}(\lambda)$, its fourth moment satisfies that

$$\mathbb{E}[W^4] = \lambda + 7\lambda^2 + 6\lambda^3 + \lambda^4.$$

This implies that

$$\mathbb{E}\left[\left(\frac{X_{i,k}}{S_k}\right)^4 \Big| S_k\right] = \alpha_i S_k + 7(\alpha_i S_k)^2 + 6(\alpha_i S_k)^3 + (\alpha_i S_k)^4 = \lambda + 7\lambda^2 + 6\lambda^3 + \lambda^4. \tag{17}$$

By the boundness, $\lambda = \alpha_i S_k \leq M$, we have (17) donimated by

$$\mathbb{E}\left[\left(\frac{X_{i,k}}{S_k}\right)^4 \Big| S_k\right] \leq M + 7M^2 + 6M^3 + M^4 \triangleq C(M). \tag{18}$$

Hence, we have

$$\sup_{S_k>0} \mathbb{E}\left[\left(\frac{X_{i,k}}{S_k}\right)^4 \Big| S_k\right] \leq C(M) < C,$$

for some constant C. By the Berry-Esseen theorem:

$$\sup_x \left|P\left(\frac{\hat{\rho}-\mu}{\sigma\sqrt{1+h}} \leq x\right) - \Phi(x)\right| \leq \frac{C_3}{\sqrt{n}}$$

where C_3 depends on standardized 3rd moments. Define $c_\alpha^* \triangleq c_\alpha/\sqrt{1+h}$. The Type I error is:

$$P(|\hat{\rho}| > c_\alpha \mid H_0) = 1 - \Phi\left(\frac{c_\alpha - \mu}{\sigma\sqrt{1+h}}\right) + \Phi\left(\frac{-c_\alpha - \mu}{\sigma\sqrt{1+h}}\right)$$

$$= \underbrace{\left[1 - \Phi\left(c_\alpha^* - \frac{\mu}{\sigma\sqrt{1+h}}\right)\right]}_{\text{Right Tail}} + \underbrace{\Phi\left(-c_\alpha^* - \frac{\mu}{\sigma\sqrt{1+h}}\right)}_{\text{Left Tail}}. \tag{19}$$

For the right tail dominance, since $\mu > 0$ and $h > 0$, we have

$$c_\alpha^* - \frac{\mu}{\sigma\sqrt{1+h}} < c_\alpha^* = \frac{\Phi^{-1}(1-\alpha/2)}{\sqrt{1+h}} < \Phi^{-1}(1-\alpha/2).$$

By monotonicity of Φ, we can derive that the right tail excesses $\alpha/2$ by

$$1 - \Phi\left(c_\alpha^* - \frac{\mu}{\sigma\sqrt{1+h}}\right) > 1 - \Phi\left(\Phi^{-1}(1-\alpha/2)\right) = \alpha/2. \tag{20}$$

Then, for the left tail bound, by Taylor's expansion, we have

$$\Phi\left(-c_\alpha^* - \delta\right) = \Phi(-c_\alpha^*) - \delta\phi(c_\alpha^*) + O(\delta^2),$$

where $\delta = \frac{\mu}{\sigma\sqrt{1+h}} > 0$. Thus, we have

$$\Phi\left(-c_\alpha^* - \frac{\mu}{\sigma\sqrt{1+h}}\right) > \Phi(-c_\alpha^*) - \delta\phi(c_\alpha^*). \tag{21}$$

Combining (21) and (20), we have

$$P(|\hat{\rho}| > c_\alpha) > \alpha/2 + \alpha/2 - \delta\phi(c_\alpha^*) = \alpha - \delta\phi(c_\alpha^*).$$

By the convexity of Φ on $(-\infty, 0]$, we have

$$\alpha/2 - \Phi(-c_\alpha^* - \delta) = \Phi(c_\alpha^* + \delta) - \Phi(c_\alpha^*) \geq \delta\phi(c_\alpha^* + \delta).$$

Moreover, since $\phi(c_\alpha^* + \delta) < \phi(c_\alpha^*)$ for $\delta > 0$, we have

$$\delta\phi(c_\alpha^*) < \delta\phi(c_\alpha^* + \delta) \leq \alpha/2 - \Phi(-c_\alpha^* - \delta).$$

which concludes that

$$P(|\hat{\rho}| > c_\alpha) > \alpha - \delta\phi(c_\alpha^*) > \alpha.$$

□

REMARK 1. *The combined effects of positive bias and variance inflation distort the distribution of $\hat{\rho}$, shifting its mass toward the tails beyond the nominal critical values. This dual mechanism inflates Type I error rates in hypothesis testing*
