# Supplementary material for: Library size-stabilized metacells construction enhances co-expression network analysis in single-cell data
Source: PLoS Comput Biol. 2025 Nov 13;21(11):e1013697. doi: 10.1371/journal.pcbi.1013697 (PMC12626273; doi:10.1371/journal.pcbi.1013697)
Supplement: S1 Table — Values represent the coefficient of variation (standard deviation divided by the mean), indicating the degree of variability in library sizes within each metacell type. (PDF) [file pcbi.1013697.s002.pdf]

	LSMetacell	hdWGCNA	Metacell2	MetaQ	SEACells	SuperCell	Primary
EN	0.099	0.765	0.231	0.385	0.583	0.973	0.555
IN	0.010	0.747	0.211	0.362	0.441	0.962	0.504
OC	0.080	0.476	0.135	0.270	1.370	0.746	0.364
AC	0.109	0.518	0.161	0.269	3.139	0.869	0.450
MC	0.158	0.512	0.169	0.273	1.484	0.867	0.469

S1 Table. Variability in library size distribution of metacells generated by different methods across major cell types. Values represent the coefficient of variation (standard deviation divided by the mean), indicating the degree of variability in library sizes within each metacell type.
