# Supplementary material for: Library size-stabilized metacells construction enhances co-expression network analysis in single-cell data
Source: PLoS Comput Biol. 2025 Nov 13;21(11):e1013697. doi: 10.1371/journal.pcbi.1013697 (PMC12626273; doi:10.1371/journal.pcbi.1013697)
Supplement: S2 Table — Each cell displays the p-value from the statistical test evaluating whether the method in the row exhibits significantly higher Zsummary scores than the method in the column. Empty cells indicate comparisons that were either not applicable or not performed. (PDF) [file pcbi.1013697.s003.pdf]

	LSMetacell	hdWGCNA	Metacell2	MetaQ	SEACells	SuperCell	Primary
LSMetacell	——	0.002	0.007	0.001	0.006	0.189	0.009
hdWGCNA	0.998	——	0.908	0.324	0.471	0.934	0.703
Metacell2	0.923	0.093	——	0.055	0.120	0.583	0.203
MetaQ	0.999	0.678	0.946	——	0.520	0.949	0.872
SEACells	0.994	0.531	0.881	0.482	——	0.859	0.718
SuperCell	0.813	0.062	0.419	0.005	0.106	——	0.117
Primary	0.991	0.299	0.799	0.129	0.283	0.884	——

S2 Table. Results of one-sided Wilcoxon rank-sum tests (left > right) comparing Zsummary scores between pairs of metacell construction methods presented in Fig 2. Each cell displays the p-value from the statistical test evaluating whether the method in the row exhibits significantly higher Zsummary scores than the method in the column. Empty cells indicate comparisons that were either not applicable or not performed.
