# Supplementary material for: Library size-stabilized metacells construction enhances co-expression network analysis in single-cell data
Source: PLoS Comput Biol. 2025 Nov 13;21(11):e1013697. doi: 10.1371/journal.pcbi.1013697 (PMC12626273; doi:10.1371/journal.pcbi.1013697)
Supplement: S3 Table — The results demonstrate a near-quadratic scaling of runtime with cell count, which aligns with its theoretical O(N2) complexity. (PDF) [file pcbi.1013697.s004.pdf]

Number of Single Cells	100	500	1000	5000	10000	15000
Time (sec.)	0.34	1.59	8.01	333.50	2188.22	6069.33

S3 Table. The table shows the LSMetacell algorithm's runtime on a standard laptop (64 GB RAM, Windows 11 OS) using datasets of varying sizes. The results demonstrate a near-quadratic scaling of runtime with cell count, which aligns with its theoretical O(N^2) complexity.
