# Supplementary material for: Library size-stabilized metacells construction enhances co-expression network analysis in single-cell data
Source: PLoS Comput Biol. 2025 Nov 13;21(11):e1013697. doi: 10.1371/journal.pcbi.1013697 (PMC12626273; doi:10.1371/journal.pcbi.1013697)
Supplement: S2 Fig — We generated a synthetic single-cell dataset in which genes possess no intrinsic pairwise correlation (see Fig 1 Methods) and applied seven metacell-building algorithms: hdWGCNA, Metacell2, SEACells, SuperCell, MetaQ, Primary, and LSMetacell. After normalization within each metacell set, all pairwise Pearson correlations between genes were recalculated; their frequency distributions are displayed above. To quantify differences in noise suppression, two-sided Wilcoxon signed-rank tests were conducted between every distribution and the LSMetacell-derived distribution (our reference). P-values are reported directly on the plots. (PDF) [file pcbi.1013697.s006.pdf]

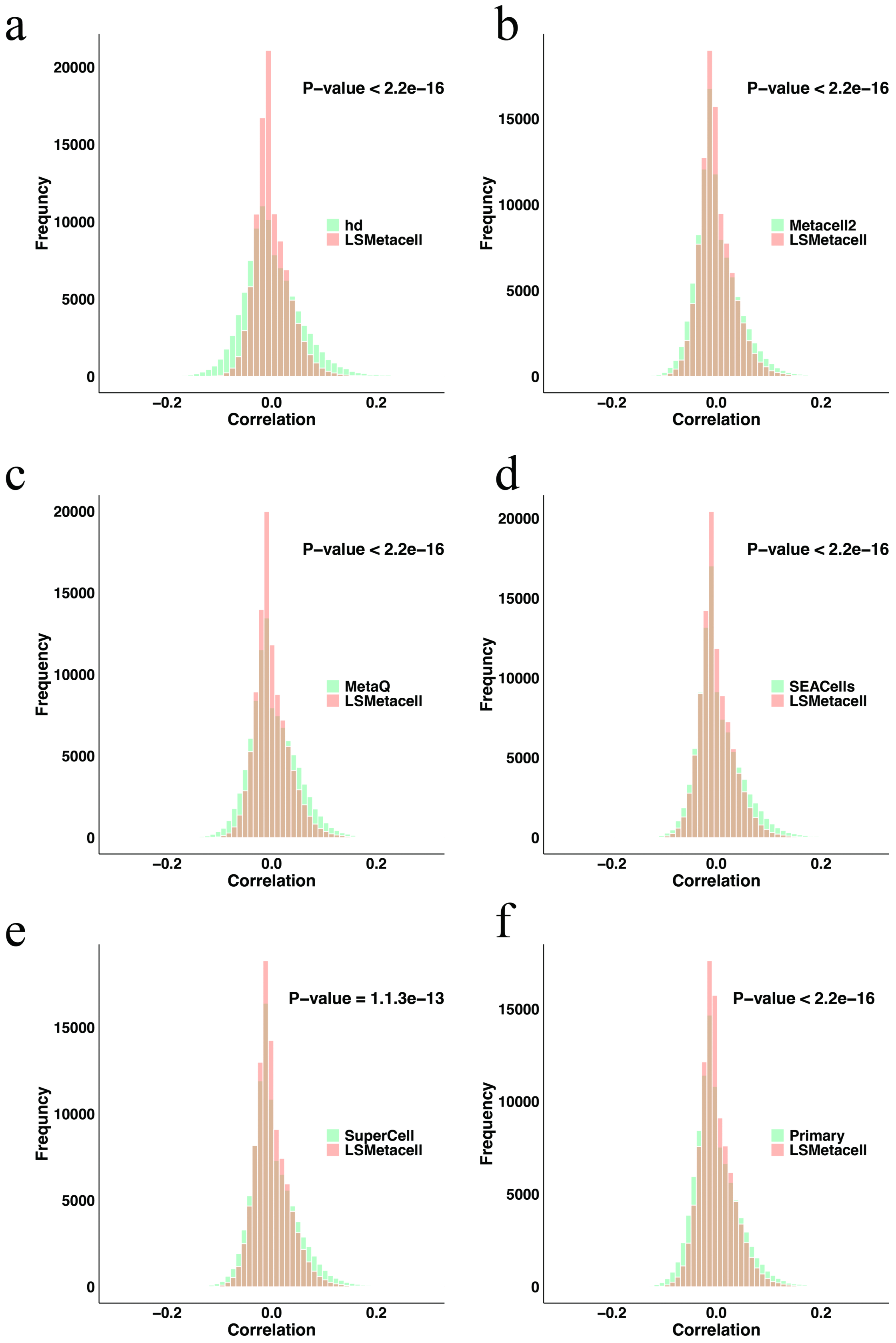


**S2 Fig.** Benchmarking metacell algorithms under a synthetic null-correlation dataset. We generated a synthetic single-cell dataset in which genes possess no intrinsic pairwise

correlation (see Fig 1 Methods) and applied seven metacell-building algorithms: hdWGCNA, Metacell2, SEACells, SuperCell, MetaQ, Primary, and LSMetacell. After normalization within each metacell set, all pairwise Pearson correlations between genes were recalculated; their frequency distributions are displayed above. To quantify differences in noise suppression, two-sided Wilcoxon signed-rank tests were conducted between every distribution and the LSMetacell-derived distribution (our reference). P-values are reported directly on the plots.
