# Supplementary material for: Library size-stabilized metacells construction enhances co-expression network analysis in single-cell data
Source: PLoS Comput Biol. 2025 Nov 13;21(11):e1013697. doi: 10.1371/journal.pcbi.1013697 (PMC12626273; doi:10.1371/journal.pcbi.1013697)
Supplement: S3 Fig — To ensure comparability across methods, the modules identified by each algorithm were aligned with those from LSMetacell using Fisher’s exact test to assess significant overlaps. Thus, modules labeled as MCgreen, MCred, MCyellow, MCblue, MCturquoise, MCblack, MCbrown, MCpink, MCpurple, MCyellow, and MCmagenta in each method correspond significantly to their counterparts in LSMetacell. Any additional modules identified by a method that are not listed above do not exhibit consistent correspondence across the other algorithms. (PDF) [file pcbi.1013697.s007.pdf]

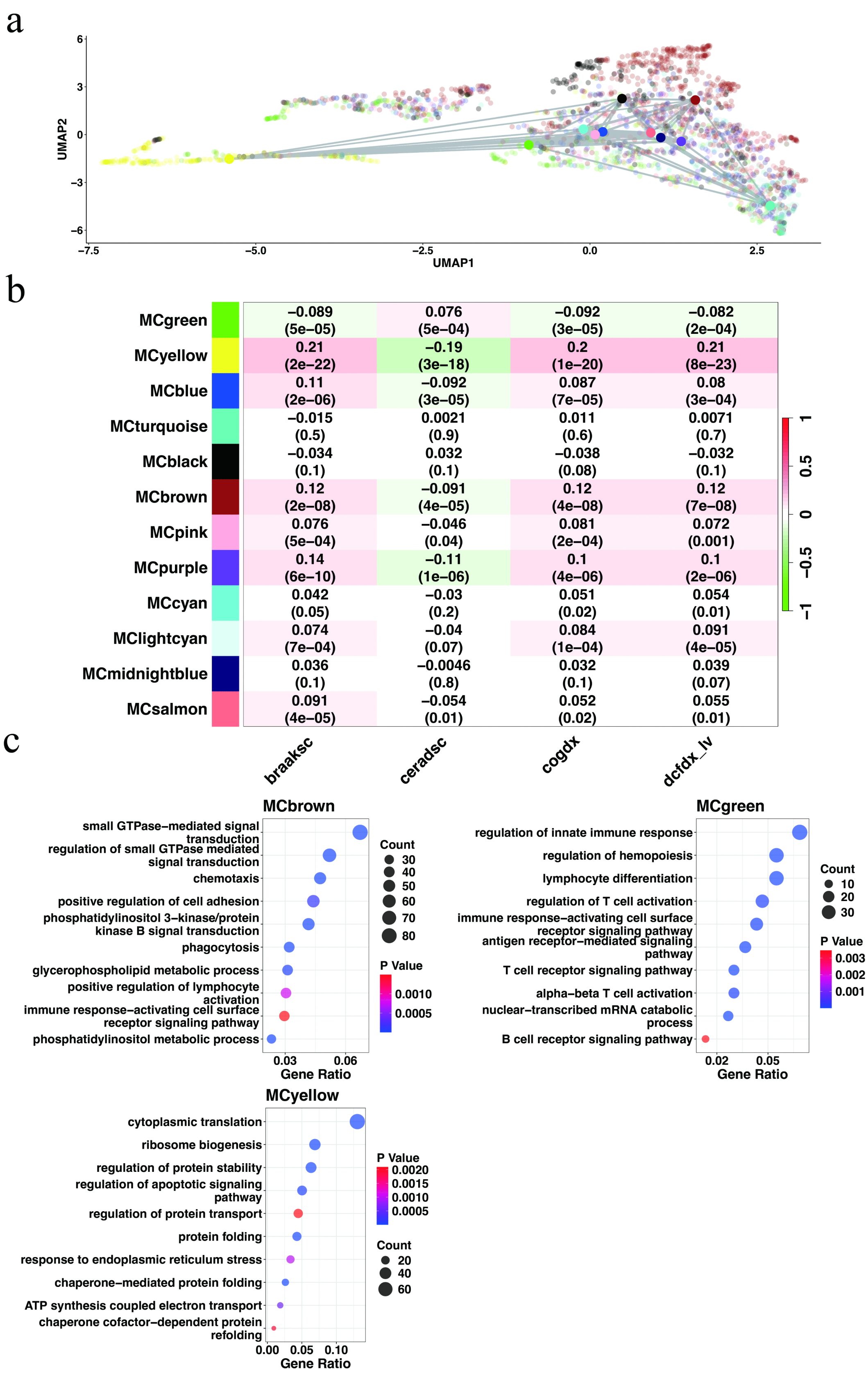

S3 Fig. Microglia gene co-expression modules identified by hdWGCNA employing the same analysis and presentation panels as in Fig 3 (see this figure for a detailed caption). To ensure

comparability across methods, the modules identified by each algorithm were aligned with those from LSMetacell using Fisher's exact test to assess significant overlaps. Thus, modules labeled as MCgreen, MCred, MCyellow, MCblue, MCturquoise, MCblack, MCbrown, MCpink, MCpurple, MCyellow, and MCmagenta in each method correspond significantly to their counterparts in LSMetacell. Any additional modules identified by a method that are not listed above do not exhibit consistent correspondence across the other algorithms.
