# Supplementary material for: Library size-stabilized metacells construction enhances co-expression network analysis in single-cell data
Source: PLoS Comput Biol. 2025 Nov 13;21(11):e1013697. doi: 10.1371/journal.pcbi.1013697 (PMC12626273; doi:10.1371/journal.pcbi.1013697)
Supplement: Attachment — Submitted filename: Response to reviewers_zhb.pdf [file pcbi.1013697.s015.pdf]

Dear Editor,

On behalf of my co-authors and myself, we would like to express our sincere gratitude for the opportunity to revise our manuscript. We deeply appreciate the time and effort you have devoted to reviewing our paper, as well as the thoughtful and constructive suggestions and feedback you provided. Your insightful comments have been invaluable in helping us improve the quality of our work. We have carefully considered all of the suggestions and have tired to address each one as thoroughly as possible. In the following section, we provide a point-by-point response to the reviewers' comments.

Sincerely

Haibin Zhu

Reviewers' comments:

Reviewer #1:

In the context of scRNA-seq, the authors address the well-known issue that normalizing library sizes through linear scaling introduces bias into estimated gene-gene correlations across single cells. As a remedy, they propose combining meta-cell formation with explicit control for library size variation. Specifically, they introduce a heuristic greedy algorithm that aggregates similar cells into meta-cells until the total count within each meta-cell reaches a predefined target library size. Through extensive validation experiments, they demonstrate that this approach effectively reduces bias in gene-gene correlation estimates, while preserving biological signal.

The idea is simple yet convincing. The validation experiments are generally convincing. Nevertheless, I would like to raise several points that could help further improve the manuscript.

Major comments:

1. The authors emphasize that their method controls for library size, unlike other approaches. However, it is important to acknowledge that standard library size normalization by scaling also “controls” library size, just in a different way. In my view, the key distinction lies in how variability across genes is handled. Scaling adjusts all gene counts within a (meta-)cell by the same factor. When different cells have different scaling factors, this uniform scaling across

genes artificially inflates gene-gene correlations. In contrast, meta-cell formation aggregates counts from multiple cells, preserving more gene-specific variability: some genes may increase their counts by, say, 10%, while others only by 5%. This heterogeneity reduces (or even eliminates) spurious correlations while still approximately equalizing library sizes across meta-cells. I suggest that the authors make this conceptual point more explicit in the manuscript.

We thank the reviewer for this insightful comment and for clearly articulating the crucial conceptual distinction between standard library size normalization and our meta-cell-based approach. We fully agree with the reviewer's perspective that while both methods aim to address library size differences, the key advantage of our metacell aggregation lies in its ability to preserve gene-specific variability and thereby mitigate spurious correlations introduced by per-cell scaling factors.

We appreciate this valuable suggestion and have revised the manuscript to explicitly clarify this conceptual point. The discussion now includes a more detailed explanation of how meta-cell formation controls for library size while better preserving biological covariation structure, in direct contrast to standard scaling methods. (revised manuscript line 311-325)

2. I believe it is also important to note that the generated meta-cell data cannot fully eliminate false correlations, as the data remain approximately compositional. One would still expect to observe the characteristic negative correlations across genes that arise in compositional data: if one gene is more highly expressed in a meta-cell, other genes must have correspondingly lower counts to maintain the fixed total library size. Were such negative correlations still observed in the meta-cell data?

We thank the reviewer for raising this critical point regarding the inherent compositional nature of transcriptomic data and its effect on spurious correlations, even after meta-cell generation. The reviewer is absolutely correct that meta-cell data, while reducing technical noise, remains approximately compositional due to the constraints of fixed library size per meta-cell. We appreciate the opportunity to clarify this issue.

When preparing the analysis in Supplementary Figure 2, we observed that a certain degree of negative correlation persists in the LSMetacell constructed metacell data, which is particularly associated with highly expressed genes, though notably reduced compared to other methods.

We have further expanded this discussion in the revised manuscript to acknowledge this limitation more explicitly (revised manuscript line 365-371). We thank the reviewer again for emphasizing this important conceptual aspect.

3. Figure 1: The main conclusion, that increasing variance in library size leads to inflated gene-gene correlations, is clearly supported. However, I am curious why the authors chose to present the bias as a function of mean gene expression, and how they interpret the specific shape of the resulting curves. Furthermore, I would appreciate an explanation for the small but consistent positive bias observed even under constant library sizes. What is the origin of this residual bias?

We thank the reviewer for their insightful comments and positive assessment that our main conclusion is clearly supported by Figure 1. We agree that a deeper explanation of the figure's details would strengthen the manuscript. We have revised the manuscript to address their questions as follows:

a. Reasons for Plotting Bias Against Mean Gene Expression: This is a standard diagnostic in genomics because the statistical properties of sequencing data are highly dependent on gene abundance. Gene expression data is heteroskedastic, meaning the variance and bias depend on the mean. Lowly expressed genes behave very differently from highly expressed genes. They are more affected by technical noise, sampling effects such as dropouts, and have lower signal-to-noise ratios. Plotting the bias against the mean allows us to visualize this dependency and demonstrate that the inflation of correlations is not uniform across all genes but disproportionately affects specific gene populations.

b. Interpretation of the Curve Shape: At low expression, genes have very noisy measurements. Many of their measured counts are zeros due to the dropouts. When the library size fluctuates, a gene might get a count of 0 in a small library and 1 or 2 in a large library. This behavior, driven by library size rather than true biology, creates strong but spurious correlations between pairs of lowly expressed genes. At mid-range expression, gene expressions are more reliably detected. Their signal is stronger relative to the technical noise. While still affected by library

size variance, the effect is less pronounced because their expression is not fluctuating around zero. At high expression, a different effect can come into play, i.e., the compositionality. These genes make up a substantial fraction of the total library. If the library size increases, their counts increase to maintain that fraction, forcing them to correlate with each other.

c. Origin of the Residual Bias under Constant Library Sizes: An interesting observation is the small but consistent bias in correlation estimates even under a constant library size. This residual bias likely originates from the inherent compositional nature of sequencing data and the statistical properties of zero-inflated counts. Because the total number of molecules in a cell is finite, gene expression measurements are not fully independent. Furthermore, applying standard correlation metrics like Pearson to discrete, zero-inflated count data can introduce artifacts. Shared zeros between two sparsely expressed genes can artificially inflate their calculated correlation, leading to the observed small positive bias from zero.

We thank the reviewer for prompting us to clarify this important nuance.

These additions provide the requested context and interpretation for Figure 1 and significantly improve the manuscript.

4. Figure 2: The authors write that "LSMetacell demonstrated higher and stable module preservation." I believe a more nuanced discussion is needed here, as this claim does not appear to hold uniformly across all cell types shown in the figure.

We are grateful to the reviewer for this insightful comment regarding the need for a more nuanced interpretation of module preservation across cell types. We agree that our original assertion was overly generalized.

In revising our analysis, we recognized that the limited number of modules per cell type, typically around 10, rendered within-type statistical comparisons underpowered. Importantly, since preservation statistics, e.g., Z-summary, are measures of modular robustness that is independent of cell type, we were able to combine these values across all cell types into a single pooled analysis. This approach provides a globally representative and statistically powerful assessment of preservation strength.

By aggregating preservation statistics in this manner, we generated a robust dataset that allowed for rigorous hypothesis testing. The updated results, now presented in Figure 1 and Supplementary Table 1, clearly demonstrate that LSMetacell yields generally higher and more consistent preservation metrics compared to alternative methods.

5. The manuscript refers to a dual “optimization” strategy underlying their algorithm. Since no formal optimization problem is actually solved, I recommend rewording this to avoid overstating the methodological rigor.

We thank the reviewer for this precise and constructive comment regarding our use of the term "optimization." We agree that this wording may overstate the methodological formalism of our approach, as our procedure does not involve solving a defined objective function in the strict mathematical sense.

In response to this feedback, we have revised the manuscript throughout to replace "optimization" with refinement. We are grateful to the reviewer for prompting this clarification, which has improved the precision of our methodological description.

5. On line 179, the authors write: “We found that the coefficient of variation of meta-cells obtained by different methods was in the order of hdWGCNA > metaQ > primary > metacell2 > LSMetacell should be interpreted...” Although this is part of the results section, a brief comment on the scientific significance of this observation would be helpful for the reader.

We thank the reviewer for this helpful suggestion. The coefficient of variation (CV) is a key metric for evaluating the technical robustness and stability of meta-cells. A lower CV indicates greater consistency in library sizes and transcriptional profiles across meta-cells, which is crucial for minimizing technical noise and improving the reliability of downstream analyses. The complete CV values for all methods and datasets are provided in Supplementary Table 2. In direct response to the reviewer’s comment, we have added the following sentence to the results section (revised manuscript line 186-188):

"Notably, LSMetacell achieved the lowest coefficient of variation, indicating superior stability of the generated meta-cells, which is a property essential for reducing noise in downstream analyses (Supplementary Table 2)."

6. In Algorithm 1, I believe that the x' in the denominator of the formula on line 21 should be $w_{x'}$.

We sincerely thank the reviewer for their meticulous reading. The reviewer is absolutely correct. The x' in the denominator should indeed be $w_{x'}$, representing the weight of the neighboring cell x'. This was a typographical error in the initial submission. We have corrected it in the revised manuscript.

Reviewer #2

1. The authors generated null datasets in which genes are not co-expressed and evaluated the level of gene co-expression at the single-cell level, for different library size variations across cells. The authors should show that their method performs well on the null datasets, i.e. minimal biases should be observed after building metacells on the null datasets.

We thank the reviewer for this important comment. In response, we have performed additional analysis using null (under the condition variance=1 in Fig. 1a, representing a common and practical level of library size variation).

As demonstrated in Supplementary Figure 2, LSMetacell introduced minimal spurious correlation in the resulting metacells compared to all other benchmark methods, confirming its ability to effectively suppress technical.

These results have been added to the Results section (revised manuscript line 162-165) of the revised manuscript. We appreciate the reviewer's feedback, which has helped us further strengthen the validation of our method.

2. Also, the analysis based on the null datasets shows an increase in averaged gene correlations when variations in library sizes increase. However, the levels of correlations are really small (Pearson coef < 0.02). It is difficult to evaluate the impact of such correlations on downstream analyses and no one would ever consider seriously such low (spurious) correlations.

We thank the reviewer for this thoughtful comment. We agree that the mean values of the spurious correlations are indeed very small. However, the central issue lies not in the mean but in the systematic change in the distribution of these correlations, a substantial increase in their variance. We add a new result for illustration in Supplementary Figure 1. This increase in variability directly elevates the false positive risk in co-expression network inference, as more correlations spuriously exceed significance thresholds by chance.

Furthermore, in the supplementary note1 we provided a mathematical proof demonstrating that the variance of the null correlation distribution increases proportionally with the variance of library size across cells. This theoretical result confirms that even small systematic biases can distort the overall correlation structure, making an impact to the downstream biological conclusions.

3. The authors should evaluate metacells qualities based on standard metrics used in the metacell field (e.g. compactness, separation, size distribution, purity, etc.). It would also be useful to project the metacells in the single-cell space to see whether metacells are well distributed over the single cell space.

We thank the reviewer for raising this important point regarding the evaluation of metacell quality using standard metrics. We agree that assessments of compactness, separation, and purity are valuable in many metacell applications.

In the revised manuscript, we have included an evaluation of metacell size distribution (Supplementary Table 1), which aligns directly with the key goal of our method—to control for library size variation during metacell construction. However, as the reviewer perceptively notes, our method is specifically designed for application within predefined and homogeneous cell types—rather than for general metacell construction across heterogeneous populations—with a primary emphasis on generating metacells with stabilized library sizes. This focus necessarily involved certain trade-offs in traditional metrics such as compactness and separation, as optimizing for library size stability can sometimes conflict with spatial compactness in transcriptional space. This design choice makes LSMetacell particularly suited for co-expression analysis within predefined cell types, rather than for general-purpose metacell applications such as unknown cell state discovery. We recognize this as a limitation in terms of broader applicability, and expand it in the Discussion section (revised manuscript line 373-391).

We appreciate the reviewer's feedback, which has helped us more clearly define the scope and strengths of our method.

4. In Table 1: the number of gene pairs with significant PPI enrichment for LSMetacell and the primary method are relatively close. The authors should test whether these differences are significant or not. Statistics indicating the significance of the differences observed between the methods benchmarked should also be added to Figure 2.

We thank the reviewer for this insightful suggestion. We agree that statistical validation between methods is essential for a rigorous comparison.

a. For Table 1, since the underlying STRING background score remains consistent within each cell type, we performed a paired Wilcoxon test to compare the number of significantly enriched PPI pairs between LSMetacell and each benchmark method across multiple cell types. The resulting p-values have been added to the caption of Table 1, confirming that the improvements with LSMetacell are statistically significant.

b. For Figure 2, given that preservation statistics (e.g., Z-summary) reflect modular robustness which independent of cell type origin, we combined these values across all cell types into a pooled global analysis. This allowed us to perform rigorous statistical testing between methods. We have updated Figure 2 accordingly and included the corresponding p-values in Supplementary Table 2.

We appreciate the reviewer's suggestion, which has strengthened the statistical validity of our comparative analysis.

5. The benchmark of LSMetacell against published methods is not comprehensive: well-known methods need to be included such as SEACells (Persad et al. Nature biotechnology, 2023) and SuperCell (Bilous et al. BMC bioinformatics, 2022).

We sincerely thank the reviewer for this constructive suggestion to broaden the scope of our benchmarking. We agree that the inclusion of additional well-established methods—such as SEACells (Persad et al., 2023) and SuperCell (Bilous et al., 2022)—would significantly strengthen the comparative analysis and offer readers a more comprehensive evaluation.

In response, we have performed new benchmarking experiments against both SEACells and SuperCell. The results, now included in the revised manuscript (see Figure 2, Table 1, and Supplementary Figure 2), demonstrate that LSMetacell consistently performs favourably across multiple key metrics when compared to these methods, including co-expression module preservation, protein-protein interaction enrichment, and minimal bias on null data.

6. In the implementation of LSMetacell (R package on github), it seems that there is no possibility to link a metacell to the single cells they contain. This is critical for the evaluation of metacells quality (including the metrics mentioned in the comment n°3).

We sincerely thank the reviewer for this valuable technical feedback. In direct response, we have updated the lib_stabilized_metacells and lib_stabilized_metacells_by_group functions in the LSMetacell R package to include a new option return_cell_index. When set to TRUE, the function returns a list with the following components:

meta_cells: the gene expression matrix of metacells.

meta_index: an integer vector that maps each original cell to its corresponding metacell ID

The updated implementation is available in the latest version on GitHub, and the documentation and vignettes have been revised to reflect this new feature. We greatly appreciate the reviewer's suggestion, which significantly improves the practical utility and evaluability of our tool.

7. This algorithm might be computationally heavy for large datasets. The authors should mention the resources needed to build metacells with LSMetacells on datasets with various sizes.

We thank the reviewer for raising this important point regarding computational efficiency. To provide a practical assessment of the computational requirements for LSMetacell, we performed runtime benchmarks on a conventional laptop computer (Windows OS, 64 GB RAM) using datasets of varying sizes (Supplementary Table 3).

We have added these in the discussion part (revised manuscript line 396-400). We appreciate this comment, which helps improve the transparency and usability of our method.

8. The authors applied LSMetacell to a brain dataset and identified biologically meaningful cell-type-specific co-expression networks. However, the authors do not show whether these networks could also be found using the algorithms that do not consider library size in the metacell construction (or directly from the single-cell data without considering metacells). Here again, much more extensive benchmark is needed

We thank the reviewer for this insightful comment. We agree that a more extensive benchmarking is necessary to rigorously evaluate whether the identified biologically meaningful networks are uniquely enabled by library size stabilization, or could also be recovered by other methods.

In response, we have performed additional comparative analyses applying benchmark metacell methods to the same brain dataset. It is important to note that we did not include direct single-cell co-expression analysis in this comparison. Due to the extreme sparsity of the single-cell data, fewer than 2,000 genes passed our expression filter (expressed in at least 10% of cells within any given cell type), rendering the resulting gene set too limited for meaningful and robust co-expression analysis. This severe sparsity makes the single-cell-level network unsuitable for a fair and functionally informative comparison with the more complete and stable metacell-based networks.

The results, now included as Supplementary Figures 3–8, demonstrate that although general co-expression patterns were consistently identified across all benchmarked metacell methods—supporting the reliability of our comparative metacell pipeline evaluation—LSMetacell revealed two biologically specialized modules with stronger relevance to Alzheimer's disease (AD) pathogenesis: an adaptive immune-associated MEgreen module and a centrally connected MEred module in microglia, enriched in RNA metabolism and other core microglial functions. These findings have been incorporated into the Results section of the revised manuscript (revised manuscript line 252-255 and line 266-269). We thank the reviewer for this suggestion, which has significantly strengthened the validation of our method.

9. In the discussion, the authors mention that one of the limitation of the method is that cell types have to be defined before using LSMetacells to build metacells, this point should be emphasized in the method section. It was indeed not clear to me that metacells should be built within each cell type.

We thank the reviewer for highlighting this important aspect of our method. We fully agree that the requirement for pre-defined cell types should be clearly emphasized in the Method section to avoid potential misunderstandings regarding the appropriate application of LSMetacell

In response to this comment, we have revised the Method section (revised manuscript 459-462). to explicitly state that metacell construction must be performed separately within each pre-annotated and homogeneous cell type.

10. On GitHub the example of the readme file cannot be run, the “dpfc” object is not defined. The authors should consider using publicly available dataset such as the Seurat pbmc data.

We sincerely thank the reviewer for highlighting this issue in our GitHub documentation. We have updated the example code and now provide a fully reproducible workflow using the publicly available PBMCSCA dataset (accessible via the SeuratData package), which ensures better transparency, accessibility, and reproducibility.

The revised README and associated scripts have been updated on our GitHub repository. We are grateful to the reviewer for this valuable suggestion, which has greatly enhanced the usability and reproducibility of our tool.

11. The quality of the figures needs to be substantially improved. Many of them are just unreadable due ot catastrophic choices of font sizes, axis names,… As reviewer, we spend lots of time evaluating papers, so a minimal efforts to make Figures readable and consistent would be much appreciated

We sincerely apologize for the substandard quality of the figures in our initial submission. We fully agree that clear and professionally presented figures are essential for both the review process and the readability of the final manuscript, and we regret that our figures did not meet this standard, thus unnecessarily complicating your review. We have increased the font sizes for all labels, axis names, and legends to ensure readability. We hope they now meet the required standards and make the evaluation of our work significantly easier. We are grateful for your patience and for holding us to a higher standard of presentation.
