# Supplementary material for: Library size-stabilized metacells construction enhances co-expression network analysis in single-cell data
Source: PLoS Comput Biol. 2025 Nov 13;21(11):e1013697. doi: 10.1371/journal.pcbi.1013697 (PMC12626273; doi:10.1371/journal.pcbi.1013697)
Supplement: Attachment — Submitted filename: response to reviewers .docx [file pcbi.1013697.s014.docx]

Dear Editor,

On behalf of my co-authors, I thank you for the opportunity to revise our paper. We appreciate the time and effort you have dedicated to reviewing our manuscript and for the constructive feedback provided. Your valuable insights have been instrumental in enhancing the quality of our work. The authors have carefully considered the comments and tried our best to address every one of them. Below, we provide a point-by-point response to the comments received.

Sincerely

Haibin Zhu

1. Cite and/or compare your approach for construction of metacells with existing methods that have been designed for that purpose using the same benchmarking you do already in the paper:

- MetaCell: analysis of single-cell RNA-seq data using K-nn graph partitions. Baran et al. Gen Biol 2019

- Building and analyzing metacells in single-cell genomics data. Bilous et al. Mol Sys Biol 2024

- MetaQ: fast, scalable and accurate metacell inference via single-cell quantization. Li et al. Nature Communciations 2025

Thank you for your insightful suggestion. In our revised manuscript, we have now incorporated citations to the key methodological papers on metacell construction—including MetaCell (Baran et al., 2019), MetaCell2 (Ben-Kiki et al., 2022), and MetaQ (Li et al., 2025)—as well as the comprehensive review by Bilous et al. (2024). To ensure a rigorous comparison, we benchmarked our method against both MetaCell2 and MetaQ using the same datasets and evaluation metrics. Our results demonstrate that our method achieves superior performance in terms of:

1. Higher and more stable module preservation scores

2. Improved protein-protein interaction (PPI) enrichment scores

These findings indicate that stabilizing library size variation in metacells significantly enhances co-expression analysis.

2. Your argumentation is that the library size of the metacells is an important factor to control in the creation of metacells. In your approach LSMetacell you do this as described in Algorithm 1. In order to prove your argument on the level of the benchmark that you use, it would be useful to create an alternative implementation of LSMetacell, where you do not control for library size at the time of aggregation, but simply control for the number of metacells for example. Then you should check if the module preservation is negatively affected by leaving out library size control.

Thank you for your valuable suggestion. We now included a primary version of LSMetacell , We implemented a primary version of LSMetacell that aggregates cells while maintaining only the number of metacells, without applying explicit library size control. Our benchmarking revealed that omitting library size control negatively impacts module preservation, reinforcing the necessity of this step. Interestingly, even the primary version of LSMetacell exhibited some reduction in library size variation compared to some other methods, highlighting its inherent robustness, though the full implementation delivered significantly better performance.
