# Supplementary material for: Library size-stabilized metacells construction enhances co-expression network analysis in single-cell data
Source: PLoS Comput Biol. 2025 Nov 13;21(11):e1013697. doi: 10.1371/journal.pcbi.1013697 (PMC12626273; doi:10.1371/journal.pcbi.1013697)
Supplement: Attachment — Submitted filename: Response to reviewers.docx [file pcbi.1013697.s016.docx]

Dear Editor,

On behalf of my co-authors and myself, I would like to express our sincere gratitude for the opportunity to revise our manuscript. I am especially grateful to you and the reviewers for the careful reading, insightful comments, and generous time devoted to helping us improve the clarity and visual quality of our work. All issues raised in the editorial summary have been addressed point-by-point below.

Sincerely

Haibin Zhu

Editor Comments

Please make sure that all figures are vector graphics and are of high enough resolution for the main and supplemental figures.

All figures have been re-generated and passed PLOS’s NAAS tool for print size, resolution, etc.

Also make sure that font sizes between subfigures are comparable. Currently there is quite a difference in font size comparing Figure 1,2 and 3.

We adjusted all text elements across Figures 1–3 and all supplementary figures.

Please rename the y-axis of Figure 2 as something easier to understand, such as Z-summary statistic.

The y-axis of Figure 2 has been relabelled Z-summary.

For Figure 3b, why's the CREAD score abbreviated as Ceradsc and not as Creadsc. Is this a typo?

Thank you for pointing out this important error. This was indeed a typo. We have corrected it to “CERAD score ” .

In Figure 3B and 3C the names for modules are different, because in B its the eigengenes (MEyellow) and in C the modules (MC_yellow). Please add a description of this difference to the figure caption, such that readers that do not know WGCNA well can follow.

We thank the Editor for highlighting this potential source of confusion. To prevent any ambiguity, we have now standardized all module labels in both Figure 3B and 3C under the prefix “MC” (Microglia-derived Co-expression module). In panel B, these MC modules (e.g., MCyellow) represent the module eigengenes of this module employed for correlation analyses, whereas in panel C the identical gene sets are used for GO enrichment. Consequently, the same MC identifier refers to the same microglial module; only the analytical context differs (eigengene versus gene list).

Reviewers' comments

1. Figures are still of very low quality with many plots being highly pixelized (not only in the pdf, also in the tif files). No effort has been made to avoid large empty spaces, points being cut by axis (e.g., Figure 3c), axis names reflecting internal variable in the code (“Zsummary.pres”), lack of axis, etc., etc. I was very clear in my previous report that Figures needed to be improved, and it looks like the authors could not care less about this point. This is utterly disappointing.

Thank you for your candid feedback. We apologise for the shortcomings in our previous figure files and for any impression that your earlier comments had been overlooked.

(1) All figures have been regenerated as vector-based PDFs and re-exported as high-resolution TIFFs; each file passed the PLOS NAAS check.

(2) We rearranged the figures to avoid large empty spaces now and truncated data points have been corrected in Figure 3c and supplementary figures.

(3) Axis name in figure 2 has been replaced; complete coordinate axes are now drawn for every plot.

2. Lines 186-188 The authors mention that “LSMetacell achieved the lowest coefficient of variation, indicating superior stability of the generated meta-cells”. I would clarify in the main text that the coefficient of variation was computed based on library size (as described in the supplementary file) and thus the stability statement refers to library size stability.

Thank you for your suggestion. We have amended line 190.

“Notably, LSMetacell achieved the lowest coefficient of variation, indicating superior **library size stability** of the generated meta-cells, which is a property essential for reducing noise in downstream analyses.”

3. In the discussion, lines 386-388 the authors mention that “in addition to library size control, other factors such as high compactness and separation of metacells may also play important roles in obtaining biologically meaningful co-expression networks”. Metacells of good quality should have low compactness and high separation not high compactness and separation.

Thank you for pointing out this important error. We have revised the sentence (line 400):

“These observations suggest that, in addition to library size control, other factors such as **low compactness and high separation of metacells** may also play important roles in obtaining biologically meaningful co-expression networks.”

4) In response to my 4th comment in the first round of revision, the authors say that they completed Table 1 by performing a “paired Wilcoxon test to compare the number of significantly enriched PPI pairs between LSMetacell and each benchmark method across multiple cell types”. However, I see only one pvalue in the legend and not a pvalue for each comparison.

The legend now explicitly states that paired Wilcoxon test was performed (LSMetacell vs. all benchmark methods across cell types), hence the identical P-value (0.031) for every comparison. We added (line 200):

“Paired Wilcoxon test comparing LSMetacell with all benchmark methods across cell types **yielded P = 0.031 for every comparison**.”
